# Monetary Health Co-Benefits and GHG Emissions Reduction Benefits: Contribution from Private On-the-Road Transport

**DOI:** 10.3390/ijerph18115537

**Published:** 2021-05-21

**Authors:** Je-Liang Liou, Pei-Ing Wu

**Affiliations:** 1 The Center for Green Economy, Chung-Hua Institution for Economic Research, Taipei 10617, Taiwan; jlliou@cier.edu.tw; 2 Department of Agricultural Economics, National Taiwan University, Taipei 10617, Taiwan

**Keywords:** benefit matrix, benefit transfer method, benefit per ton, health, social cost of carbon, value of statistical life

## Abstract

This is the first study to provide a systematic monetary benefit matrix, including greenhouse gas (GHG) emissions reduction benefits and air pollution reduction health co-benefits, for a change in on-the-road transport to low-carbon types. The benefit transfer method is employed to estimate the social cost of carbon and the health co-benefits via impact pathway analysis in Taiwan. Specifically, the total emissions reduction benefits from changing all internal combustion vehicles to either hybrid electric vehicles, plug-in hybrid electric vehicles, or electric vehicles would generate an average of USD 760 million from GHG emissions reduction and USD 2091 million from health co-benefits based on air pollution reduction, for a total benefit of USD 2851 million annually. For a change from combustion scooters to light- or heavy-duty electric scooters, the average GHG emissions reduction benefits would be USD 96.02 million, and the health co-benefits from air pollution reduction would be USD 1008.83 million, for total benefits of USD 1104.85 million annually.

## 1. Introduction

Different response measures for greenhouse gas (GHG) mitigation, such as a carbon tax, emissions trading schemes, subsidies, performance standards, energy efficiency improvement, or carbon capture, etc., by switching all economic activities to low-carbon types have been developed to reduce the impact of climate change on humans, and different approaches are being adopted in many countries. Thus, a response measure is the general and broad term for a mitigation measure. Regardless of which approach is employed, resources are required. The social cost of carbon (SCC) is an indicator used to assess the performance of response measures in relation to climate change at the global scale. The SCC is a top-down monetary measurement used to compute the damage caused to a society by one ton of GHG emissions or the benefit that a society gains from one ton of GHG emissions reduction [1,2,3]. The SCC comprehensively measures damage to human health, property, agricultural productivity due to floods or drought, the ecological environment, and many other aspects from one ton of GHG emissions in a monetary magnitude. If GHG emissions are reduced by one ton, then the benefit will result in the avoidance of damage. To evaluate the scope of impact, a large and complete model is required.

Different mitigation mechanisms for controlling GHG emissions will not only reduce global GHG emissions but also eliminate local air pollution and positively impact human health [4]. The benefits from the concurrent elimination of local air pollution and the reduction in GHG emissions are termed co-benefits. This term first appeared in the third assessment report of the Intergovernmental Panel on Climate Change (IPCC) to indicate the benefits beyond those of GHG emissions reduction arising from the implementation of any mechanism for such reduction [4]. Co-benefits are also called ancillary benefits or side benefits, and these three terms include the benefits originating from other mechanisms concurrently implemented along with GHG emissions reduction schemes. The term co-benefit was not unified and universally used until the IPCC report in 2018. Shukla and Chaturvedi [5] noted that co-benefits will not occur automatically with any GHG emissions reduction mitigation policy; they require a comprehensive and detailed plan.

Among all sectors that emit GHGs, the transportation sector emits a higher share than the agriculture, construction, waste, land use or forest sectors [6]. Different types of transport emit different types of air pollutants in addition to GHG emissions. Previous endeavors have explored efficiency improvement when adopting different plug-in electric vehicles at household levels in the United States, with a better design of a low-voltage grid for electric vehicles in Europe, or in the transition to electric vehicles under the implementation of tax incentives in Iceland [7,8,9]. However, these efforts mainly focus on comparing the level of GHG emissions reduction or cost savings from the use of different electric vehicles. Peters et al. [10], in a very recent study related to the study here, explored the climate and health co-benefits trade-off under six different electric vehicle adoption scenarios, where the total CO_2_ emissions reduction and premature deaths represent the outcomes.

To the best of our knowledge, no study has simultaneously evaluated the GHG emissions reduction benefits and health co-benefits from switching to electric transportation in comparable monetary terms. As different types of transport require different fuels and emit varying amounts of GHGs and other types of air pollutants, the exploration and measurement of health co-benefits for the transportation sector has been identified as an important research area [11,12,13]. It is essential and important to evaluate the benefits of reducing GHG emissions and the health co-benefits achieved through the reduction in PM_2.5_ and its precursors, NO_x_ and SO_x_, under different transport uses.

Data from the Environmental Protection Administration, Taiwan (Taiwan EPA), indicate that GHG emissions from the transportation sector account for 13% of total emissions [14]. Among these, on-the-road transport vehicles and scooters account for 51% and 12%, respectively. Other forms of transport represent the remaining 37%, i.e., different sized trucks represent 30% and public transport represents 7%. The transportation sector contributes approximately 30% of the air pollution concentration. The data further indicate that vehicle and scooter transport contribute approximately 27.4% and 21.1% of the air pollution concentration, respectively [15]. However, the GHG emissions not only depend upon the composition of sectors but also upon the government energy development goal. The government in Taiwan has set the target of 50% of energy coming from the source of natural gas, 30% from coal, and 20% from renewable energy by 2025.

The main objective of this study is to monetize the benefits of GHG emissions reduction and the health co-benefits of air pollution reduction for the private on-the-road transportation sector in Taiwan. This is the first study to provide a comprehensive monetary benefit matrix generated from the GHG emissions reduction benefits and health co-benefits for changes in one type of private-on-the-road vehicle or heavy/light scooter transport to the other type. The information provided in this study is essential for implementing subsidies to encourage private vehicles or scooters/motorcycles (scooters, hereafter) to change to low-carbon transport. This study contributes to the energy policy and public health literature a complete process for the evaluation of GHG emissions reduction benefits and health co-benefits for all sectors other than the transport sector.

Moreover, the idea of displaying the benefit matrix evaluated in this study makes clear policy implications about the difference between GHG emissions reduction benefits and health co-benefits when switching to different energy uses of private on-the-road transports. The benefit transfer method is employed to estimate the SCC and health co-benefits via the impact pathway approach to accomplish the above objective. The benefit transfer method aims to bring the capabilities of previous nationwide or international data, simulation results, or estimated equations into full play, either temporally and/or spatially. As with the impact pathway approach, the physical pathways for emissions from air, water, or soil sources are the core of the analysis.

The remaining sections are arranged as follows. Section 2 presents the background and literature review. Section 3 presents the methods of monetizing GHG emissions reduction and health impacts. Section 4 presents the data sources and the computations for all kinds of monetary benefits. Section 5 presents the results and offers a discussion. Section 6 provides the conclusions and policy implications of this study.

## 2. Literature Review

The scope of co-benefits includes economic, environmental, and social dimensions. Specific aspects include the health of the human body, food security, ecological service systems, sustainable development, and technology change [16]. New studies on co-benefits have consistently been published over the past 10 years [17], but most studies only identified the aspects of co-benefits and stated their importance, presented the physical type of indicators, assessed the spatial change in mortality, or classified the estimation methods for all types of physical indicators [9,16,18,19]. Karlsson et al. [20] is one exception that cited various types of health co-benefits measured in USD arising from the reduction in NH_3_, SO_x_, NO_x_, PM, and NMVOC in different countries due to different GHG emissions reduction and mitigation mechanisms.

Because exposure to polluted air increases the risk of heart attack, stroke, lung cancer, and acute or chronic respiratory disease, health co-benefits are consistently the focus of attention. In particular, the health co-benefits from PM-related pollution have become one of the most important items in evaluations of the impact of GHG emissions reduction [11,21]. Furthermore, exposure might increase medical expenditure and cause mortality [22]. According to research by the World Bank and the Institute for Health Metrics and Evaluation [23], 5.5 million premature deaths were caused by polluted air, accounting for approximately one tenth of the total deaths worldwide and leading to a monetized health loss of approximately USD 5100 billion in 2013.

Updated information provided by [24] shows that exposure to PM_2.5_ causes a burden of 977,140 deaths from ischemic heart disease globally and a USD 21.93 million loss in 2017 from monetized disability-adjusted life years. Although human health is normally the major and direct concern when assessing PM_2.5_ pollutants, Schiferl and Heald [25] evaluated net crop production due to air quality changes and found that maize increased by 5.6%, wheat decreased by 3.7%, and rice increased by 4.5% globally. Although not all these impacts are negative, there is clearly a direct negative effect on human health for those whose staple food is wheat and an indirect effect for others if reallocation of food is necessary. The negative impact is also shown for studies performed for specific countries or regions, such as study done by [26] for various countries in Asia.

In the above studies, only a few of the co-benefits of GHG emissions reduction have been measured in monetary terms, particularly in combination with other measured monetary benefits, to determine through cost–benefit analysis whether a specific RM should be implemented [27]. The most frequently used monetary benefit, the SCC, is inadequate and does not fully incorporate health co-benefits. Among the different criticisms, Scovronick et al. [28] noted the deficiency in the SCC and indicated that although the integration of the Policy Analysis of the Greenhouse Effect (PAGE) by [29], the Dynamic Integrated Climate-Economy model (DICE) by [30], and the Framework for Uncertainty, Negotiation and Distribution (FUND) by [31] in the computation of the SCC implicitly or explicitly captures health damage, the diseases or health risks considered are either incomplete or out of date.

That is, the results from the integration of these three models consider mortality caused by temperature increases only and do not account for mortality caused by other factors due to climate change. Moreover, data on mortality computation by temperature increase have not been updated with the most current models. These drawbacks suggest that a complete and up-to-date estimation of the health co-benefits of GHG emissions reduction is required in addition to estimating the SCC following integration of the PAGE, FUND, and DICE models.

In addition, in previous studies, the co-benefits were only monetized for a specific country as a whole, and it is hard to use such outcomes as a guide for policy implementation because people cannot always perceive when and how the outcomes of response measures, such as carbon tax payments for GHG emissions reduction, are realized. Thus, it is necessary to monetize the potential health co-benefits based on specific behaviors to identify the benefits of GHG emissions reduction. Furthermore, due to the global nature of GHG emissions, it is reasonable to expect that if an RM involves more than one country, such as a cross-country cap-and-trade mechanism, it will be difficult, if not impossible, to reach consensus among countries through negotiation or bargaining [32]. If the health co-benefits from GHG emissions reduction for each country are already higher than the emissions reduction benefits, international coordination or cooperation may not be necessary, as the regional benefits of each country will already be sufficient to achieve ambitious nationally determined contributions [33,34].

## 3. Research Methods

**Figure 1:**
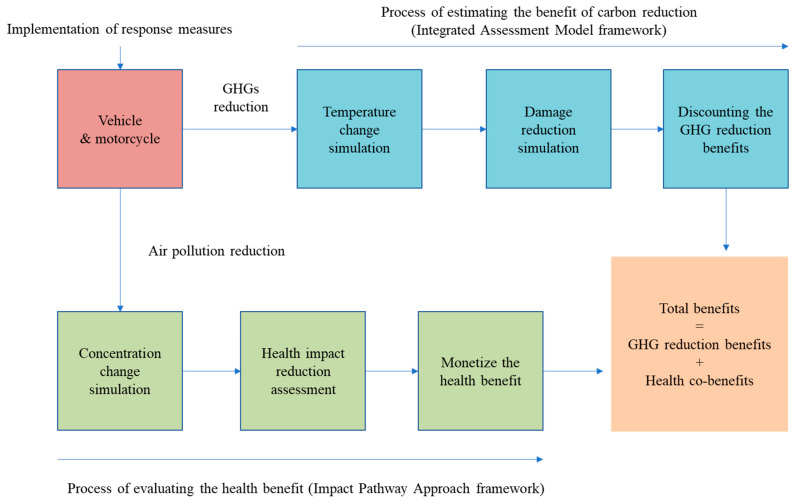
Conceptual framework for monetizing greenhouse gas (GHG) reduction benefits and health co-benefits.

There are two components of benefits when evaluating GHG emissions reduction: the monetized measurement of GHG emissions reduction and the health co-benefits of air pollution reduction. These two types of benefits must be taken into account to capture the complete benefits of GHG emissions reduction through different response measures. The conceptual framework is presented in Figure 1. For GHG emissions reduction and the health co-benefits, the benefit transfer method then generates the necessary data, preparing it for the use of monetary evaluations of environmental costs or benefits. Both the benefit transfer method and the impact pathway approach connect or integrate different disciplines, allowing them to share one another’s achievements. The following subsections explain the evaluation of each type of benefit.

### 3.1. Monetizing GHG Emissions Reduction Benefits

To include the social cost of GHG emissions and the benefits of GHG emissions reduction in its policy evaluation, the US formed an Interagency Working Group on the Social Cost of Carbon in 2010 and updated it in 2016 to monetize the social cost for each ton of GHG emissions, which is defined as the SCC [1,2]. From a methodological perspective of environmental economics for the SCC, a decline in damage is viewed as beneficial. As a result, the SCC can be used to measure the social cost of an increase in GHG emissions as well as to evaluate the benefit of GHG emissions reduction. The integrated assessment model developed by the US is basically an integration of three major climate models, i.e., PAGE, DICE and FUND.

Thus, to embrace the diversity with the scope of SCC, its outcome is simulated by the integrated assessment model using the PAGE, DICE, and FUND [2]. The fundamental purpose of these three models is to evaluate the impacts of global warming by connecting climate and the economy through a comprehensive set of dimensions. Evaluating or simulating the costs and/or benefits from GHG emissions mitigation policies or strategies adapted for climate change are crucial and essential missions of these three models. The advantage of integrated assessment model is the most extensive methodology used internationally. Moreover, it is possible to clearly connect GHG emissions reduction benefits.

The global-scale models measure the social cost of one ton of GHG emissions. The function of the integrated assessment model is to evaluate not only the climate but also the environmental impact of GHG emissions, such as sea level increase, rainfall change intensity, and extreme weather frequency. The global-scale SCC unifies the environmental impact of GHG emissions and is applied universally without differentiation by area. One ton of GHG emissions, regardless of the emission location, will cause the same damage on a global scale. These physical impacts can thus be further monetized as damage.

In other words, the rationale for using the global SCC estimated by the United States Environmental Protection Agency (US EPA) is that GHG emissions reductions internal to Taiwan will benefit any country, as GHG emissions are a global external issue. No individual country can resolve this issue independently, and the actions taken by one country will have connections to all other countries. Thus, GHG emissions reduction can be achieved by using global SCC [35,36,37]. For policy evaluation purposes, the US EPA updates the SCC every year to reflect changes in societal preferences and conditions, with the most recent version being from 2016 [38]. The SCC results from this most current version are used to estimate the monetary value of GHG emissions reduction in this study.

### 3.2. Monetizing Health Impacts

The impact pathway approach is the mainstream tool used to evaluate the health impact of air pollution elimination in monetary terms [39,40,41]. The impact pathway approach evaluation framework combines different stages and disciplines: the first stage simulates the effect of a change in air pollution emissions on pollution concentration; the second stage computes the change in medical events from that change in pollution concentration; and the final stage measures the change in medical events in monetized terms.

The advantage of the impact pathway approach is that it allows us to clearly understand the pathway of each stage from the generation of air pollution emissions to its corresponding impact. Therefore, use of the impact pathway approach requires different types of professional knowledge and modeling skills in the areas of pollution expansion, health impacts, and monetized measurement. The greatest challenge when using the impact pathway approach is the acquisition of all related coefficients. Thus, for operational purposes, some simpler and reduced forms have been proposed and applied [42,43,44,45]. The environmental benefits mapping and analysis program-community edition (BenMAP-CE) developed by the US EPA in 2003 is one of these reduced forms.

Fann et al. [42] and Fann et al. [46] applied BenMAP-CE to quantify the number of premature deaths, and chronic and acute illnesses due to PM_2.5_ and ozone from 23 sectors in the US. A reduced form is used to replicate air quality and health impacts for emissions assessments to avoid resource-intensive requirements. Reduced-form tools are often used by the US EPA to approximate complex analyses with a lower computational burden [45]. From a technological viewpoint, reduced treatment involves a uniform assumption to estimate the impact of air pollution concentration on the reduction in each unit of emissions in the first stage of the impact pathway approach. The health impact function and monetized transformation are then used to compute the health effect for one ton of emission reduction. This is called the benefit per ton (BPT) at the country level for air pollution improvement. The advantage of BPT is not only inheriting the attributes of the impact pathway approach but also reducing the operation time and resource costs in the implementation of impact pathway approach.

In the context of the BPT, the “average” at the national scale is estimated for human health impacts and monetized benefits related to air quality based on emissions of PM_2.5_ and its precursors, SO_x_ and NO_x_, as annual and 24 h PM_2.5_ concentrations [43]. Thus, the BPT provides a quick calculation of health benefits per ton of PM_2.5_ and its precursors, SO_x_ and NO_x_, at the national or regional scale for a selected year rather than for a specific location at a specific time. Since the computation of BPT requires a large amount of data, a BPT result for a typical pollutant is typically used for several years until the expansion simulation is updated. The US EPA applied regulatory impact analysis to produce the first national-level BPT report in 2013, which included PM_2.5_ and its precursors, i.e., PM_2.5_, NO_x_, and SO_x_. The second BPT report was delivered five years later in 2018 [44].

#### 3.2.1. Simulation of Emissions and Air Quality Expansion

There are three stages for computing BPT under the impact pathway approach framework, as a three-dimensional model is used to connect the relationship between emissions and pollution concentration [43,44,47,48]. To provide stable long-term data for the expansion simulation, Taiwan EPA established a support center in 2002 to assemble data and conducted the “Community Multiscale Air Quality with Decoupled Direct Method” (CMAQ-DDM) project using a three-dimensional model to provide sensitivity simulation analysis of different areas with point, line, and area PM_2.5_ emission reductions for 2016 [49]. The results from the CMAQ-DDM method applied by Taiwan EPA [14,49] provide the BPT used in this study, i.e., a nationwide average benefit from air pollution reduction.

#### 3.2.2. Health Impact Function

The second stage of the impact pathway approach computes the health impact from pollution expansion. A health impact function is thus required at this stage. The most frequently used health impact function has the following functional form Equation (1):

(1) $$\Delta y=\left(1-\exp(-\beta \Delta x)\right)\times y_{0},$$

where $y_{0}$ is the incidence rate for a specific disease and Δy is the change in the incidence rate. Δx is the change in pollutant concentration, with the amount of E measured in tons, and β is the coefficient estimated from the concentration-response function.

The expectation of a specific health incidence occurrence, ΔI, can thus be determined by the rate of occurrence of a specific health incidence change, Δy, due to a change in pollution concentration multiplied by the number in the impacted population, as in Equation (2) below:

(2) ΔI=Δy×pop,

where pop is the population affected by the specific health incidence change. Under the framework of the BPT, the result of Equation (2) is the reduction in the expected health incidence from an average one-ton reduction in air pollution.

#### 3.2.3. Monetizing Health Co-Benefits

When a change in air pollution reduces the impacted population and damage, the monetized health benefit increases accordingly, while it decreases if the opposite occurs. The monetized health benefits of air pollution include changes in morbidity and mortality. A study by [50] indicated that a decline in mortality contributes 98% of the health benefit from the reduction in air pollution, while a decline in morbidity contributes the remaining 2%, and thus this study takes mortality to represent the health benefits of air pollution improvement. The value of a statistical life (VSL) is a typical monetary indicator of health effects based on loss due to mortality [51,52], and so the BPT can be computed as Equation (3):

(3) BPT=ΔI×VSL

## 4. Data Sources and Computation of Different Monetary Benefits

To estimate the benefit of GHG emissions reduction and the health co-benefits considering different types of transport, various categories of data are required to achieve benefit computations for GHG emissions reduction, and four types of data are required for the computation of health co-benefits based on air pollution. The following subsections present each type of data and the corresponding computation and determinations for each monetary benefit, respectively.

### 4.1. Data for the Computation of GHG Emissions Reduction and Determination of the SCC

There are two procedures used to compute the monetary value of GHG emissions reduction. The conceptual framework for the benefits of GHG emissions reduction presented in Figure 1 is first to obtain the GHG emissions from each type of transport considered here and then to acquire the information for the determination of the SCC. As with the GHG and air pollution emissions, data for vehicles and scooters are taken from the report of the “Taiwan Greenhouse Gases, Regulated Emissions, and Energy Use in Transportation” model (Taiwan GREET) assessed by means of a life-cycle approach (LCA). The LCA provided by Taiwan EPA in that report includes four stages [53]: extraction, acquisition, and transportation of inputs and fuels for each type of transport and the subsequent manufacturing process; transport operation; and transport scrap treatment. The LCA in Taiwan GREET presents the full life cycle for a type of transport from raw materials to end-of-life: it includes inputs, manufacture, operation, and waste treatment processes.

The emissions of GHG and other types of air pollutants are obtained for each of these steps as an average. Thus, when the vehicles or scooters are in operation, the emissions of GHG and other air pollutants will be the product of miles traveled and the average emissions for that type of transport. Taiwan GREET has data for internal combustion vehicles with engines of 1600 to 2000 C.C. (ICEVs), hybrid electric vehicles (HEVs), plug-in hybrid electric vehicles (PHEVs), and electric vehicles (EVs). It includes scooters with 50 C.C. and 125 C.C. engines and 19.8 and 27.2 Wh/km electric scooters. The basic GHG emissions data for each type of vehicle are listed in Table 1 for computing the benefits of GHG emissions reduction.

The US EPA routinely estimates the SCC as one of its missions, and the most recent version of its report provides the SCC for each year from 2010 to 2050 [38]. The SCCs from this report under different discount rates are listed in Table 2. The selection of the discount rate for the case at hand, the 20-year real rate of return on government bonds minus the price inflation rate proposed by [39], is 2%. This rate is close to that in Table 2 under 2.5% for 2020, the year nearest to 2019. Thus, a price of USD 62 per ton is used to transform the amount of GHG emissions to monetary units for each type of transport.

### 4.2. Data for the Estimation of Health Co-Benefits from Air Pollution Reduction

For the estimation of the health co-benefits from the reduction in air pollution of PM_2.5_ and its precursors, SO_x_ and NO_x_, as prioritized by Taiwan EPA, various types of data are required to transform the amounts of air pollution to monetary health co-benefits corresponding to the conceptual framework presented in Figure 1.

#### 4.2.1. Data for Emissions of PM_2.5_ Its Precursors SO_x_ and NO_x_

The air pollution data for PM_2.5_ and its precursors SO_x_ and NO_x_ are similar to the GHG emissions given in Table 1 and taken from [54] by the Taiwan GREET assessment of the LCA. Their estimated results are presented in Table 3 for three types of air pollution and different types of vehicles and scooters. All else being equal, each magnitude is the amount of emissions for a specific type of air pollution generated by driving or riding a typical vehicle or scooter of the specified type. The emissions difference would be the increase or decrease due to a switch between vehicles or scooters. For instance, driving an ICEV one kilometer will produce 0.026 g of PM_2.5_ emissions; if an electric-type HEV is driven instead, it will produce 0.018 g PM_2.5_ emissions. Therefore, a switch from an ICEV to an HEV driving one kilometer will produce 0.008 g less PM_2.5_ emissions.

#### 4.2.2. The Effect on PM_2.5_ Concentration from a One-Ton Emissions Reduction in PM_2.5_, SO_x_, and NO_x_

The relationship between air pollution and its concentration is taken from [14,49]. The relevant reports use the CMAQ-DDM model version 10 and the “Taiwan Emission Data System” (TEDS), with 2016 as the base year. The methodology and simulation process adopted by Taiwan EPA are consistent with the report issued by the US EPA in 2011 and meet the requirements for BPT estimation. The model considers PM_2.5_ and its precursor emissions and the concentration relationship for point, line, and area simulations.

Specifically, Taiwan EPA provides a daily simulation of the impact of the emissions of PM_2.5_ and its precursors SO_x_ and NO_x_ on PM_2.5_ concentrations for January, April, July, and November using CMAQ-DDM. The average effect of these four months, which account for emissions quantity and quality, represents the performance for the whole year. Existing data have been applied extensively in policy analyses. Levy et al. [55] also used the average simulation results for CO_2_ emissions reduction from CMAQ-DDM under the Clean Power Plan to evaluate the health benefits for all residential homes in the continental US in 2013. The results of the point, line, and area simulations from the EPA report [14,49] listed in Table 4 are used for further analyses.

#### 4.2.3. Data and Computation of Health Impact Analysis

For the health impact, the change in mortality risk is used as an indicator. Equation (1) is used to compute the health impact of the change in pollution concentration. Because there is no epidemiology research outcome for the risk mortality due to PM_2.5_ in Taiwan, the two most used β coefficients for the health impact function are adopted; these are from [56,57] and listed in Table 5. The change in the incidence rate, Δy in Equation (1), can be computed from the results for the line concentration of PM_2.5_ in Table 4 and the incidence rate $y_{0}$ data from [58], listed in Table 5.

Since each coefficient has a different age coverage, the incidence occurrence, ΔI, in Equation (2) can thus be computed from the corresponding incidence rate and the population data in 2019 for each selected β coefficient. The population data are taken from [59]. Accordingly, two computed health incidence occurrences are obtained: one represents the upper bound of incidence occurrence, and the other represents the lower bound. There are thus two BPTs corresponding to each incidence occurrence, and the final BPT is the average of these.

#### 4.2.4. Data and Calculation of Monetary Health Co-Benefits

With the above data, the health co-benefits related to air pollution from PM_2.5_ and its precursors, SO_x_ and NO_x_, can be monetized. The benefit transfer method employed from a study by [60] in the estimation of *VSL* for Taiwan in 2014 accounts for the wage differentiation for different groups of people. Two other dimensions are considered to adjust for temporal differences between the years given the transfer of this value: the income difference and the price difference. According to [60], the income difference for the *VSL* can be adjusted as in Equation (4):

(4) $$VSL_{2019}=VSL_{2014}\times \left\{1+\frac{\left[\in_{w}\times (W_{2019}-W_{2014}/W_{2014})\right]}{100}\right\},$$

where $VSL_{2019}$ is the nominal *VSL* with adjustment, and $\in_{w}$ is the income elasticity computed by [60]. $W_{2019}$ and $W_{2014}$ are the monthly average income in Taiwan in 2019 and 2014, respectively.

The other adjustment is accomplished by Equation (5):

(5) $$real\_VSL_{2019}=VSL_{2019}\times (CPI_{2019}/CPI_{2014}),$$

where $real\_VSL_{2019}$ is the price-adjusted real *VSL*, and $CPI_{2019}$ and $CPI_{2014}$ are the consumer price index for 2019 and 2014, respectively, as listed in Table 6. Accordingly, $real\_VSL_{2019}$ in Equation (5) is then calculated as USD 11.787 million in 2019. This result, along with the variables and coefficients stated above, is applied to calculate the BPT for line PM_2.5_ pollution reduction for different forms of transport. The results show that the highest, lowest, and average BPTs are USD 5.24 million, USD 1.31 million, and USD 3.28 million per ton of PM_2.5_, respectively. The average BPT is used for the subsequent analyses.

## 5. Results and Discussion

### 5.1. GHG Emissions Reduction and Its Benefits for Different Forms of Transport

Before proceeding with the analyses, some background information about the average driving or riding mileage per vehicle or scooter, the number of vehicles, light-duty and heavy-duty types of scooters is required. The Directorate General of Highways states that the average annual driving mileage per vehicle is approximately 13,890 km and the average annual riding mileage per scooter is approximately 4964 km [63]. The numbers for the different types of vehicles and different types of scooters are obtained from the same source of data. These data are required to compute total annual GHG emissions per vehicle and scooter, as shown in Table 7 and Table 8. The GHG emissions reduction from changing from a traditional type of vehicle or scooter to a low-carbon vehicle or scooter can then be computed accordingly.

Since HEVs, PHEVs, and EVs are all electric, they have lower GHG emissions than an ICEV vehicle. Thus, the change in total annual GHG emissions per vehicle when changing from a traditional ICEV vehicle to an HEV type is the difference between the ICEV and HEV columns in Table 7, i.e., 29,544,604 − 16,985,889 = 12,558,715 kiloton (kt) GHG emissions reduction for such vehicle change. The vehicle change from an ICEV is not limited to any one electric-type, be it HEV, PHEV, or EV; a change from a PHEV to an EV is also possible.

The same procedure is applied to compute GHG emissions reduction from a change in scooter type. It is reasonable to assume that a change in scooter is most likely to be between equivalent sized engines, e.g., for light-duty scooters, a change between a 50 C.C. engine and a 19.8 Wh/km electric engine, and for heavy-duty types, a change between a 125 C.C. engine and a 27.2 Wh/km electric engine. However, it is also possible that a 50 C.C. combustion engine scooter owner would both change to an electric type and upgrade to a heavy-duty scooter, while it is rare but not impossible that a 125 C.C. combustion scooter owner would change to a light-duty electric type. The GHG emissions reduction for the former scooter change would be the difference between 209,835 tons and 61,145 tons, i.e., 148.69 kt, as shown in Table 8.

**Figure 2:**
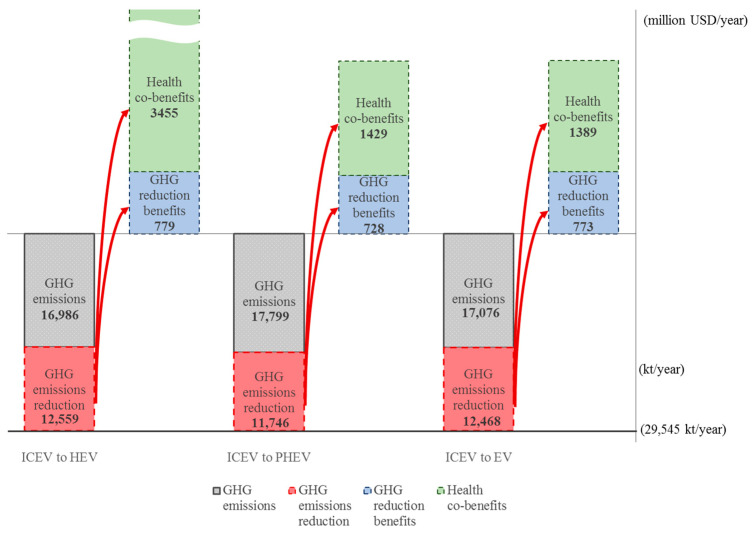
GHG reduction benefits and the health co-benefits from changing internal combustion vehicles to electric vehicles.

**Figure 3:**
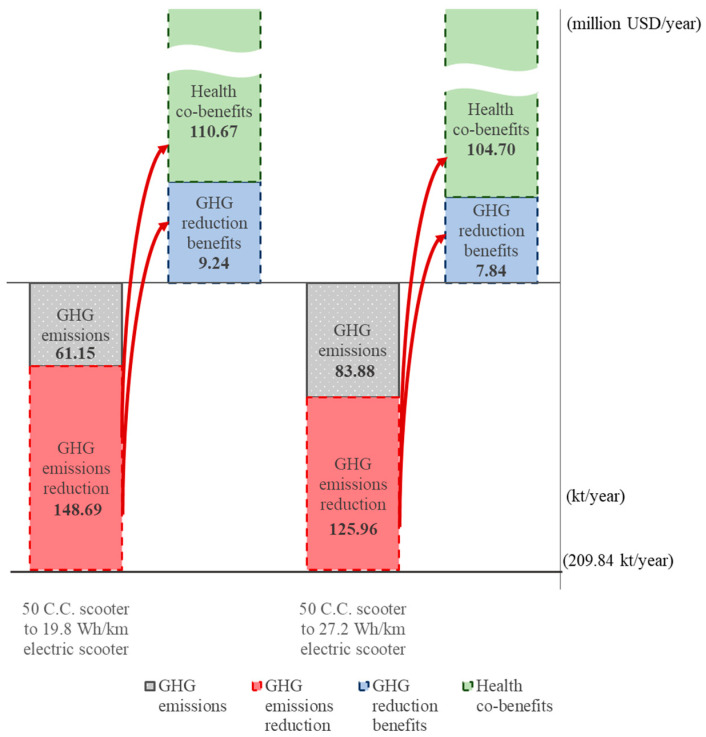
GHG emissions reduction benefits and health co-benefits from changing 50 C.C. scooters to low-carbon electric scooters.

**Figure 4:**
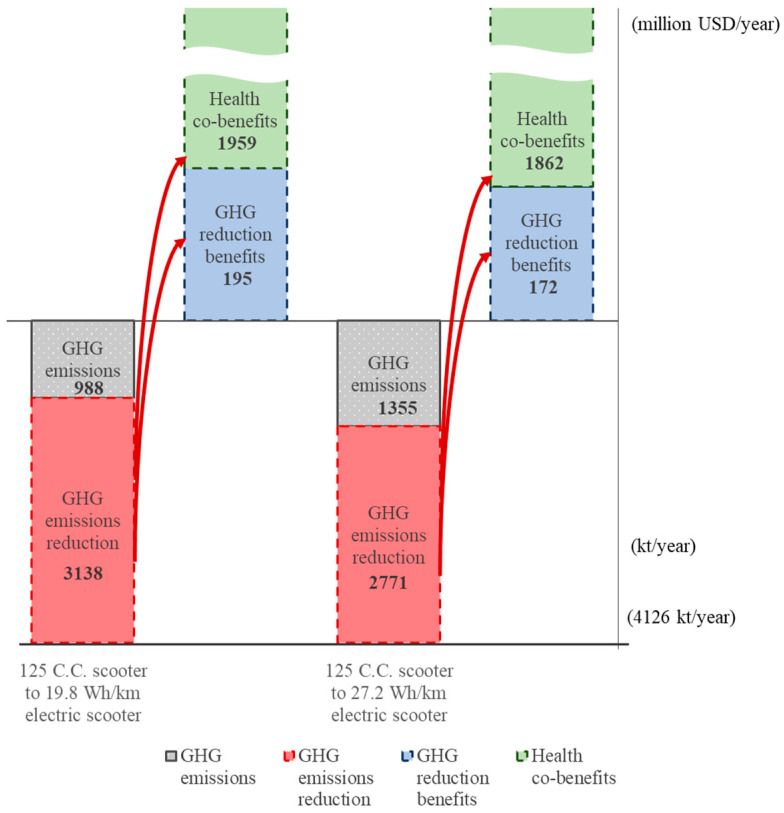
GHG emissions reduction benefits and health co-benefits from changing 125 C.C. scooters to low-carbon electric scooters.

The highest potential GHG emissions reduction for vehicles and light- and heavy-duty scooters is shown in the bottom parts of Figure 2, Figure 3 and Figure 4, respectively. The highest emissions level is 29,545 kt if all 6,501,905 vehicles remain the traditional internal combustion type. Similarly, the highest emissions are 209.84 kt and 4126 kt for light-duty and heavy-duty scooters, respectively, with no change for low-carbon scooters. The greatest GHG emissions reduction for vehicles, light-duty scooters, and heavy-duty scooters are the bars marked by the red dotted portion.

The reduction in GHG emissions estimated in this study will not only generate global benefits but also generate local reduction in air pollution. Theoretically, the total benefits of reducing certain amount of GHG will generate both global benefits and local benefits. Thus, if there is an optimal level of GHG emissions reduction to determine, this study provides information regarding the benefits for cost–benefit analysis in order to make a rational decision. Pragmatically, if only information regarding benefits is known, then the total benefits both from GHG emissions reduction and air pollution reduction can be used as an incentive for people to switch to low-carbon vehicles. Certainly, if there is only cost information, then the total benefits from GHG emissions reduction and air pollution reduction can be explained as a ceiling of the cost saved by the designated level of GHG emissions reduction.

### 5.2. Use of the Benefit Matrix

In addition to computing the amount of GHG emissions reduction for the change from internal combustion engine to electric transport as discussed above, we can further determine the monetary value of each emissions reduction to reflect the emissions reduction benefit and the associated health co-benefits. This calculation creates a benefit matrix capturing all combinations of vehicle changes, as shown in Table 9, and for 50 C.C. and 125 C.C. scooter changes, as shown in Table 10. The magnitudes in Table 9 and Table 10 are in monetary terms of USD per 1000 km (km), indicating a monetary benefit for every 1000 km of driving or riding for any either type of vehicle or scooter transport. Some magnitudes in Table 9 are explained as examples for the use of benefits in the matrix. There are USD 20.27 of GHG reduction benefits and USD 113.93 of health co-benefits, with a total of USD 134.20 per 1000 km when all ICEVs vehicles are eliminated. This is the case with the greatest GHG emissions reduction when vehicles with the largest GHG emissions are all excluded. Another case is when all ICEVs vehicles shift to one type of electric vehicle—HEVs, for instance. This will generate USD 8.62 of GHG reduction benefits and USD 38.24 of health co-benefits, with a total of USD 46.86 USD per 1000 km of driving or riding.

It is known from the results shown in Table 9 that if the change is extreme and replaces all ICEVs with an electric vehicle, it will generate the highest benefits in terms of both emissions reduction and health co-benefits. However, although these circumstances could be met, they cannot be sought; it is difficult to expect people to stop driving cars to reduce GHG emissions and increase others’ health benefits unless these behaviors and attitudes are enforced. However, it is possible to offer incentives to encourage those people currently driving ICEV-type vehicles to change to electric vehicles.

It is clear from Table 9 that positive emissions reduction benefits and health co-benefits come from the change in ICEVs to any type of electric vehicle (i.e., PHEVs, HEVs, or EVs) and from changes between specific types of electric vehicles. These magnitudes are shown in bold black or bold green in Table 9. Among these, the results systematically show that the change from an ICEV vehicle to any electric vehicle (PHEV, HEV, or EV) produces the greatest emissions reduction benefits and health co-benefits. However, some cases of a switch from one electric type to another, such as from HEVs to PHEVs or from HEVs to EVs, do not produce both positive emissions reduction benefits and health co-benefits. This is because PHEV and EV electric vehicles are all imported in Taiwan, and so the amount of GHG emissions produced by transportation and shipping must be included, causing the emissions per kilometer for these two types of vehicles to be higher than those for the HEV type.

The emissions reduction benefits and health co-benefits for all possible changes from 50 C.C. or 125 C.C. to any electric type are computed in a similar manner. The results, shown in Table 10, indicate that a change from 50 C.C. or 125 C.C. scooters to the equivalent type, 19.8 Wh/km or 27.2 Wh/km, respectively, creates reasonable emissions reduction benefits and health co-benefits. If, however, a 125 C.C. scooter is changed to a light-duty electric scooter, it will result in higher emissions reduction benefits and health co-benefits than those that change type but not engine size. Similar to the benefit matrix for vehicles, if the 50 C.C. and 125 C.C. scooters are all eliminated, this would produce the greatest benefits for emissions reduction and health.

Taking the benefit matrix values in green bold type in Table 9 and Table 10, given the GHG emissions reduction for each type of transport shown in the bottom part of Figure 2, Figure 3 and Figure 4, the corresponding GHG emission reduction benefits and health co-benefits can be computed by the information from the unit monetary benefit per 1000 km, the number of vehicles, 50 C.C. scooters and 125 C.C. scooters, and the average mileage travelled each year. The GHG emissions reduction benefits and health co-benefits are shown in the upper part of Figure 2, Figure 3 and Figure 4. It is clear that the health co-benefits are much higher than the GHG emissions reduction benefits for all types of transport. If all 6,501,905 ICEVs change to an HEV, PHEV, or EV, the health co-benefits will be 4.44, 1.96, or 1.80 times the GHG emissions reduction benefits, respectively, for an average of 2.75 times the GHG emissions reduction benefits. Similarly, if all 785,567 50 C.C. scooters and all 12,689,574 125 C.C. scooters change to either type of electric scooter, the health co-benefits generated will average 12.61 times and 10.41 times the emissions reduction benefits.

## 6. Conclusions and Policy Implications

This study accomplishes the estimation of GHG emission reduction in monetary terms and also estimates air pollutant reduction along with GHG emissions reduction in monetary term for private-on-the transport. The procedures for the estimation are certainly applicable to the other sectors when the data are available. As a result, such sound information for a comprehensive monetary measurement of GHG emissions reduction and its health co-benefits is essential for the control of GHG emissions and air pollutants emissions.

Such information is also important for tax fee determination, one of the policy designs in the control of GHG emissions. People normally have sensitive and strong feelings regarding the change in local air pollution. As the reduction in GHGs will simultaneously reduce the levels of air pollutants, it is much easier to persuade people to accept the tax fee for cutting GHG emissions. Furthermore, emissions reduction is not cost-free, and information about the benefits from switching to low-carbon private on-the-road transport is essential to encourage people to make the change. Government subsidies are always a good initiative to create a change in the desired direction. The results from this study provide information that can be used to determine an appropriate incentive for different types of private transport, the number of each type of private transport, and the amount for each subsidy. If the focus is on the GHG emissions reduction benefits and health co-benefits as this study presents, the subsidy can be equally allocated for a vehicle or a scooter switching from a traditional one to an electric type. If the subsidy reflects the transport switching in a large or in a small city, representing different densities of population, or transport switching in urban and rural areas, then different subsidies could be required to reflect these differences. Under such circumstances, the GHG emissions reduction benefit and health co-benefits for different regions or areas should be estimated accordingly.

The conventional view regarding changes in GHG emissions in the transportation sector is to pursue high emissions reduction goals. For this objective, changing all current ICEV to HEV, PHEV, and EV will reduce emissions by 42.51%, 39.76%, or 42.20%, respectively, in the case at hand. Without further information on the exact number of vehicles changing from internal combustion to an electric type, it is reasonable to take the emissions reduction of 41.49% achieved by averaging the change to each of the three electric vehicle types. Similarly, for the most commonly used form of daily transport in Taiwan, scooters, the GHG emissions decrease by 60.03% to 76.05% depending upon the original scooter type and the selected electric scooter. Since the information regarding GHG emissions reduction benefit and health co-benefits per 1000 km driving or riding is provided in a benefit matrix, the total benefit for any combination of vehicles and/or scooter switching to electric type can then be computed.

Currently, there are more than 13 million scooters in Taiwan with either 50 C.C. or 125 C.C. combustion engines, which is approximately double the number of vehicles. It is much easier to subsidize a change in scooters to an electric type. If all 125 C.C. and 50 C.C. scooters are subsidized to encourage a change to the equivalent electric type, it would generate USD 2153 million in total benefits annually from emissions reduction and health co-benefits. Similarly, the level of benefits obtained from a change in vehicles is approximately the same as providing USD 2157 million in incentives for all 6,501,905 vehicles to change to the PHEV type or USD 2162 million per year for vehicles to change to the EV type. This information can help related agencies to determine the targeted transport types and the number to subsidize.

This study accounts for health co-benefits only, as this is the most important aspect. Other co-benefits can be included if the data needed to evaluate these benefits are available and complete and methods can be determined to estimate the practicable benefits of GHG emissions reduction. Further studies can utilize the matrix constructed in this study to compute emissions reduction benefits and/or health co-benefits for any change in private on-the-road transport. The full framework of this study may also be applicable to other sectors if the corresponding data are available.

Although many efforts have been made to accomplish this study, it is inevitable that there are certain limitations for this study. As with the method of benefit transfer, there are different modes of benefit transfer. One is the average benefit transfer, another is benefit function transfer, and another is meta-transfer. What we use in the current study is average benefit transfer. The average benefit transfer could also be transferred from a set of values in the existing literature, since the estimation of VSL is highly related to the preference and socio-economic conditions of a society.

Thus, if a set of values is used as a transferred VSL, the set of values should come from the previous studies accomplished in the same country as the transferred VSL used now. However, there no other VSL has been conducted in Taiwan in the past. Thus, it is impossible to obtain a set of values to generate the transferred average VSL. Moreover, if there is a set of values, then these values are closer to the time that the transferred value can estimate. It is the best choice to use the most recent average benefit conducted in Taiwan to transfer the VSL used in this study. If it is believed that the transferred value from a set of data is more reliable than from one value, then accumulation of more VSL values is the only way one can remove such a limitation.

Moreover, since the results of this study are computed from a large amount of data, any step of data might change the final results. The results presented in this study are normally not one hundred percent certain. Thus, a good way to manage the possible uncertainty is to conduct a sensitivity analysis for the change in reasonable data and/or variables. Based upon the results presented in this study, further study can manage sensitivity analysis for the related work.

Based upon this study, further research can be expanded to discuss the possible economic impact of GHG reduction measures implemented by the transport sector on the entire economy, such as assessing how the labor market is affected by the deterioration or improvement of air pollution through changes in health risks and further assessing how the entire economy is affected. In this context, the assessment framework proposed in this study can be combined with macroeconomic impact analysis methods, such as the computable general equilibrium model or input-output model, to analyze the economic effects of the implementation of GHG emissions reduction measures in the transport sector and in all other sectors.

## Figures and Tables

**Table 1:** GHG emissions from the life-cycle approach (LCA) for different types of vehicles and scooters.

Type of Vehicle and Scooter	GHG Emissions (g/km)
Type of Vehicle	
Internal Combustion Engine Vehicle (Engine 1600~2000 C.C.) (ICEV)	327
Hybrid Electric Vehicle (HEV)	188
Plug-In Hybrid Electric Vehicle (PHEV)	197
Electric Vehicle (EV)	189
Type of Scooter	
Scooter (Engine 50 C.C.)	53.81
Electric Scooter (19.8 Wh/km)	15.68
Scooter (Engine 125 C.C.)	65.50
Electric Scooter (27.2 Wh/km)	21.51

Source: Data from [54].

**Table 2:** Estimated social cost of carbon (SCC) values under different discount rates for different years by the United States Environmental Protection Agency (US EPA).

Year	Discount Rate ^1^		
	Average at 5% Discount Rate	Average at 3% Discount Rate	Average at 2.5% Discount Rate
2010	10	31	50
2015	11	36	56
2020	12	42	62
2025	14	46	68
2030	16	50	73
2035	18	55	78
2040	21	60	84
2045	23	64	89
2050	26	69	95

Source: Data from [38]. Note: ^1^ All magnitudes are with measured in USD per ton.

**Table 3:** PM_2.5_, SO_x_, and NO_x_ emissions via LCA for different types of vehicles and scooters.

Type of Vehicle and Scooter	Pollutant Item		
	PM_2.5_ Emissions (g/km)	SO_x_ Emissions (g/km)	NO_x_ Emissions (g/km)
Type of Vehicle			
Internal Combustion Engine Vehicle (Engine 1600~2000 C.C.) (ICEV)	0.026	0.152	0.217
Hybrid Electric Vehicle (HEV)	0.018	0.161	0.129
Plug-In Hybrid Electric Vehicle (PHEV)	0.022	0.241	0.195
Electric Vehicle (EV)	0.022	0.274	0.198
Type of Scooter			
Scooter (Engine 50 C.C.)	0.0067	0.0126	0.0790
Electric Scooter (19.8 Wh/km)	0.0007	0.0079	0.0150
Scooter (Engine 125 C.C.)	0.0068	0.0153	0.0950
Electric Scooter (27.2 Wh/km)	0.0009	0.0109	0.0210

Source: Data from [54].

**Table 4:** The effect of a one-ton reduction in PM_2.5_, SO_x_ and NO_x_ on the concentration of PM_2.5_ from different sources.

Source	Types of Pollutant ^1^		
	PM_2.5_	SO_x_	NO_x_
Point	−0.00009866	−0.00001413	−0.00000500
Line	−0.00013522	+0.00141693	−0.00000629
Area	−0.00007858	−0.00002752	−0.00004108

Source: [14,49]. Note: ^1^ All pollutants are measured in units of ug/m^3^/ton.

**Table 5:** Data used to compute the expectation of mortality in the health impact analysis.

Source of Study	β Coefficient ^1^	Age Coverage	Coverage of the $\mathrm{Population}\;pop{\;}^{2}$	Incidence $\mathrm{Rate}\;y_{0}$
Krewski et al. [56]	0.005827	30–99	16,215,070	0.00568
Lepeule et al. [57]	0.013103	25–99	17,824,524	0.01015

Note: ^1^ β s are the two most commonly used in the related studies. ^2^ Taiwan’s total population in 2019 was 23,316,818.

**Table 6:** All related coefficients and data used in the estimation of the value of a statistical life (*VSL*).

Variable or Coefficient	Magnitude Used	Source of Data
Income elasticity for *VSL* ($\in_{w}$)	0.2476	[60]
Average income and salary (*W*)	$W_{2014}$: 1323.4 USD/month $W_{2019}$: 1385.6 USD/month	[61]
Consumer price index (CPI)	$CPI_{2014}$: 98.93 $CPI_{2019}$: 102.55	[62]
$VSL_{2014}$	11.785 million USD	[60]

**Table 7:** Annual GHG emissions for each type of vehicle.

Item Related to GHG Emissions	Type of Vehicle ^1^			
	ICEV	HEV	PHEV	EV
GHG emissions (g/km) (A)	327	188	197	189
Average annual driving mileage (km) (B)	13,896	13,896	13,896	13,896
Total annual GHG emissions per vehicle (ton/vehicle/year) (C) = (A)*(B)	4.54	2.61	2.74	2.63
Number of vehicles in 2019 (D)	6,501,905	6,501,905	6,501,905	6,501,905
Total annual emissions per vehicle (ton/year) (E) = (C)*(D)	29,544,604	16,985,889	17,799,043	17,076,239

Note: ^1^ For comparison, it is assumed that the average annual driving mileage and the number of vehicles is the same for the different types of vehicles.

**Table 8:** Annual GHG emission for each type of scooter.

Item Related to GHG Emissions	Type of Scooter ^1^			
	Scooter (Engine 50 C.C.)	Electric Scooter (19.8 Wh/km)	Scooter (Engine 125 C.C.)	Electric Scooter (27.2 Wh/km)
GHG emissions (g/km) (A)	53.81	15.68	65.5	21.51
Average annual riding mileage (km) (B)	4964	4964	4964	4964
Total annual GHG emission per scooter (ton/scooter/year) (C) = (A)*(B)	0.27	0.08	0.33	0.11
Number of scooters in 2019 (D)	785,567	785,567	12,689,574	12,689,574
Total annual emissions per scooter (ton/year) (E) = (C)*(D)	209,835	61,145	4,125,913	1,354,937

Note: ^1^ For comparison, it is assumed that the average annual riding mileage is the same for all types of scooters, that there is the same number of 50 C.C. engine scooters and 19.8 Wh/km electric scooters, and that there is the same number of 125 C.C. scooters and 27.2 Wh/km electric scooters.

**Table 9:** Benefit matrix for GHG emission reduction and the corresponding health co-benefits from changing vehicle types.

Replacement Vehicle Type or Elimination of the Original Vehicle	Original Type of Vehicle ^1^							
	ICEV		HEV		PHEV		EV	
	GHG Reduction Benefits	Health Co-Benefits	GHG Reduction Benefits	Health Co-Benefits	GHG Reduction Benefits	Health Co-Benefits	GHG Reduction Benefits	Health Co-Benefits
ICVE	0 ^a^	0 ^a^	−8.62 ^a^	−38.24 ^a^	−8.06 ^a^	−15.82 ^a^	−8.56 ^a^	−15.37 ^a^
	(0) ^a^		(−46.86) ^a^		(−23.88) ^a^		(−23.93) ^a^	
Eliminating driving ICEV	20.27 ^b^	113.93 ^b^	---	---	---	---	---	---
	(134.20) ^a^		(---)		(---)		(---)	
HEV	8.62 ^c^	38.24 ^c^	0 ^a^	0 ^a^	0.56 ^a^	22.42 ^a^	0.06 ^a^	22.87 ^a^
	(46.86) ^c^		(0) ^a^		(22.98) ^a^		(22.93) ^a^	
Eliminating driving HEV	---	---	11.66 ^b^	75.69 ^b^	---	---	---	---
	(---)		(87.35) ^b^		(---)		(---)	
PHEV	8.06 ^c^	15.82 ^c^	−0.56 ^a^	−22.42 ^a^	0 ^a^	0 ^a^	−0.50 ^a^	0.45 ^a^
	(23.88) ^c^		(−22.98) ^a^		(0) ^a^		(−0.05) ^a^	
Eliminating driving PHEV	---	---	---	---	12.21 ^b^	98.11 ^b^	---	---
	(---)		(---)		(110.32) ^b^		(---)	
EV	8.56 ^c^	15.37 ^c^	−0.06 ^a^	−22.87 ^a^	0.50 ^a^	−0.45 ^a^	0 ^a^	0 ^a^
	(23.93) ^c^		(−22.93) ^a^		(0.05) ^a^		(0) ^a^	
Eliminating driving EV	---	---	---	---	---	---	11.72 ^b^	98.56 ^b^
	(---)		(---)		(---)		(110.28) ^b^	

Note: “---“ in the table indicates that a vehicle cannot be eliminated because it is not currently driven. ^1^ All magnitudes are measured in units of USD/1000 km. The numbers in parentheses are the sum of GHG reduction benefits and health co-benefits. ^a^ Either the GHG emission reduction benefits or the health co-benefits is positive or that their sum is positive. ^b^ GHG reduction benefits and health co-benefits from completely eliminating the vehicles that are currently driven. ^c^ GHG reduction benefits and health co-benefits from the more likely change in vehicles from traditional to electric types.

**Table 10:** Benefit matrix of GHG emission reduction and corresponding health co-benefits from changing scooter types.

Replacement Scooter Type or Elimination of Original Scooter	Original Type of Scooter ^1^							
	Scooter (Engine 125 C.C.)		Scooter (Engine 50 C.C.)		Electric Scooter (27.2 Wh/km)		Electric Scooter (19.8 Wh/km)	
	GHG Reduction Benefits	Health Co-Benefits	GHG Reduction Benefits	Health Co-Benefits	GHG Reduction Benefits	Health Co-Benefits	GHG Reduction Benefits	Health Co-Benefits
Scooter (engine 125 C.C.)	0 ^a^	0 ^a^	−0.72 ^a^	−2.71 ^a^	−2.73 ^a^	−29.56 ^a^	−3.09 ^a^	−31.09 ^a^
	(0) ^a^		(−3.43) ^a^		(−32.29) ^a^		(−34.18) ^a^	
Eliminating the original 125 C.C. scooter	4.06 ^b^	35.53 ^b^	---	---	---	---	---	---
	(39.39) ^b^		(---)		(---)		(---)	
Scooter (engine 50 C.C.)	0.72 ^a^	2.71 ^a^	0 ^a^	0 ^a^	−2.01 ^a^	−26.85 ^a^	−2.37 ^a^	−28.38 ^a^
	(3.43) ^a^		(0) ^a^		(−28.86) ^a^		(−30.75) ^a^	
Eliminating the original 50 C.C. scooter	---	---	3.34 ^b^	32.82 ^b^	---	---	---	---
	(---)		(36.16) ^b^		(---)		(---)	
Electric scooter (27.2 Wh/km)	2.73 ^c^	29.56 ^c^	2.01 ^c^	26.85 ^c^	0 ^a^	0 ^a^	−0.36 ^a^	−1.53 ^a^
	(32.29) ^c^		(28.86) ^c^		(0) ^a^		(−1.89) ^a^	
Eliminating the original electric scooter (27.2 Wh/km)	---	---	---	---	1.33 ^b^	5.97 ^b^	---	---
	(---)		(---)		(7.30) ^b^		(---)	
Electric scooter (19.8 Wh/km)	3.09 ^c^	31.09 ^c^	2.37 ^c^	28.38 ^c^	0.36 ^a^	1.53 ^a^	0 ^a^	0 ^a^
	(34.18) ^c^		(30.75) ^c^		(1.89) ^a^		(0) ^a^	
Eliminating the original electric scooter (19.8 Wh/km)	---	---	---	---	---	---	0.97 ^b^	4.44 ^b^
	(---)		(---)		(---)		(5.41) ^b^	

Note: “---” in the table indicates that a scooter cannot be eliminated because it is not currently driven. ^1^ All magnitudes are measured in units of USD/1000 km. The numbers in parentheses are the sum of GHG reduction benefits and health co-benefits. ^a^ Either the GHG emission reduction benefits or health co-benefits is positive or that their sum is positive. ^b^ GHG reduction benefits and health co-benefits from completely eliminating the scooters that are currently driven. ^c^ The GHG reduction benefits and health co-benefits from the more likely change in scooters from traditional to the electric type.

## Data Availability

Data is not publicly available, though the data may be made available on request from the corresponding author.
